# Supplementary material for: A large-scale machine learning study of sociodemographic factors contributing to COVID-19 severity
Source: Front Big Data. 2023 Mar 24;6:1038283. doi: 10.3389/fdata.2023.1038283 (PMC10080051; doi:10.3389/fdata.2023.1038283)
Supplement: Supplementary file 1 [file Data_Sheet_1.PDF]

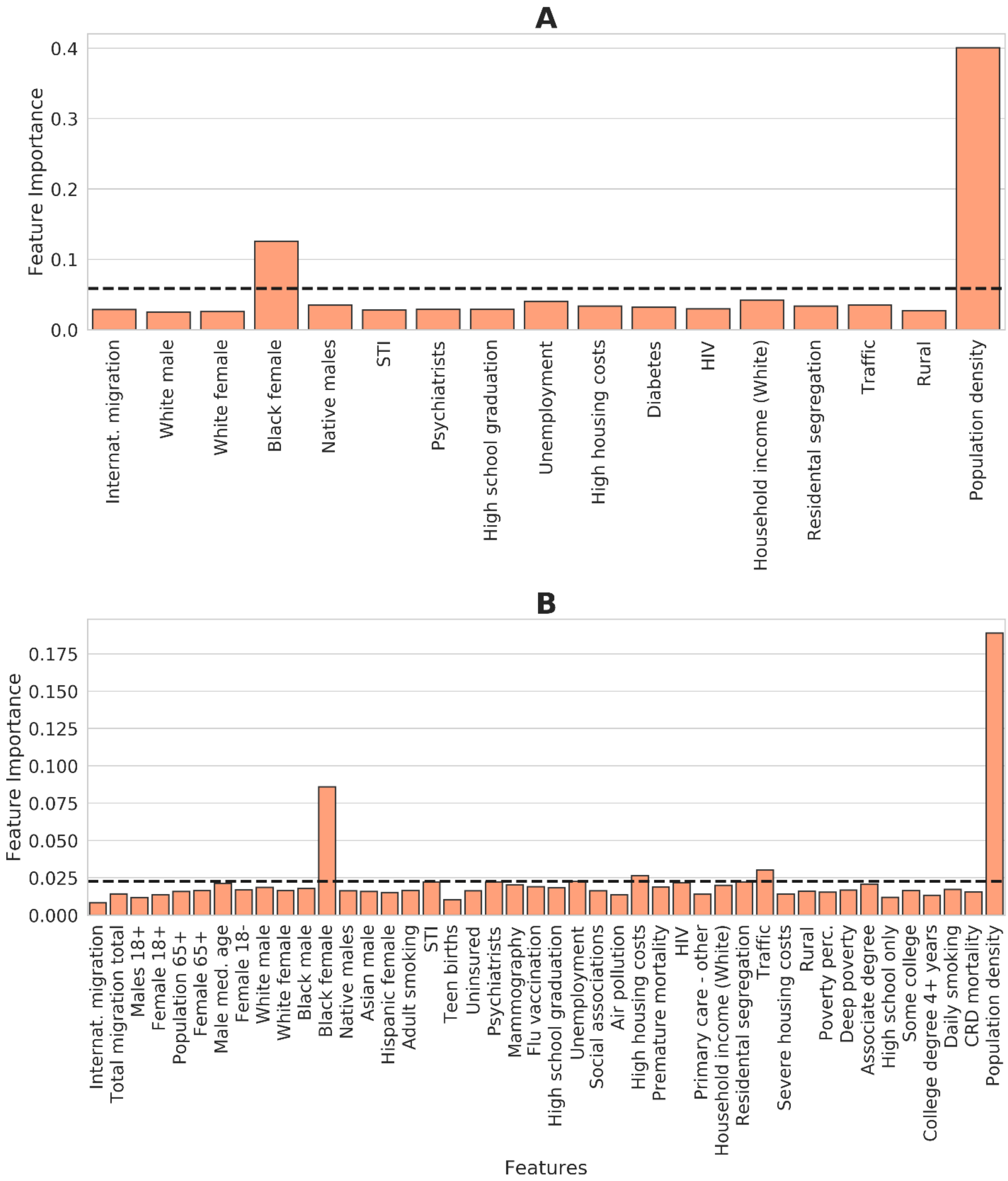

Supplementary Figure 1: Estimated variable importance in relaxed Random Forest and XGBoost methods. A) Random Forest and B) XGBoost methods are implemented in the relaxed procedure, with the variables selected after the first round indicated on the horizontal axes. Estimated variable importance is shown on the vertical axis. The horizontal line indicates the standard threshold for the significant predictors (corresponding to the predictor mean).

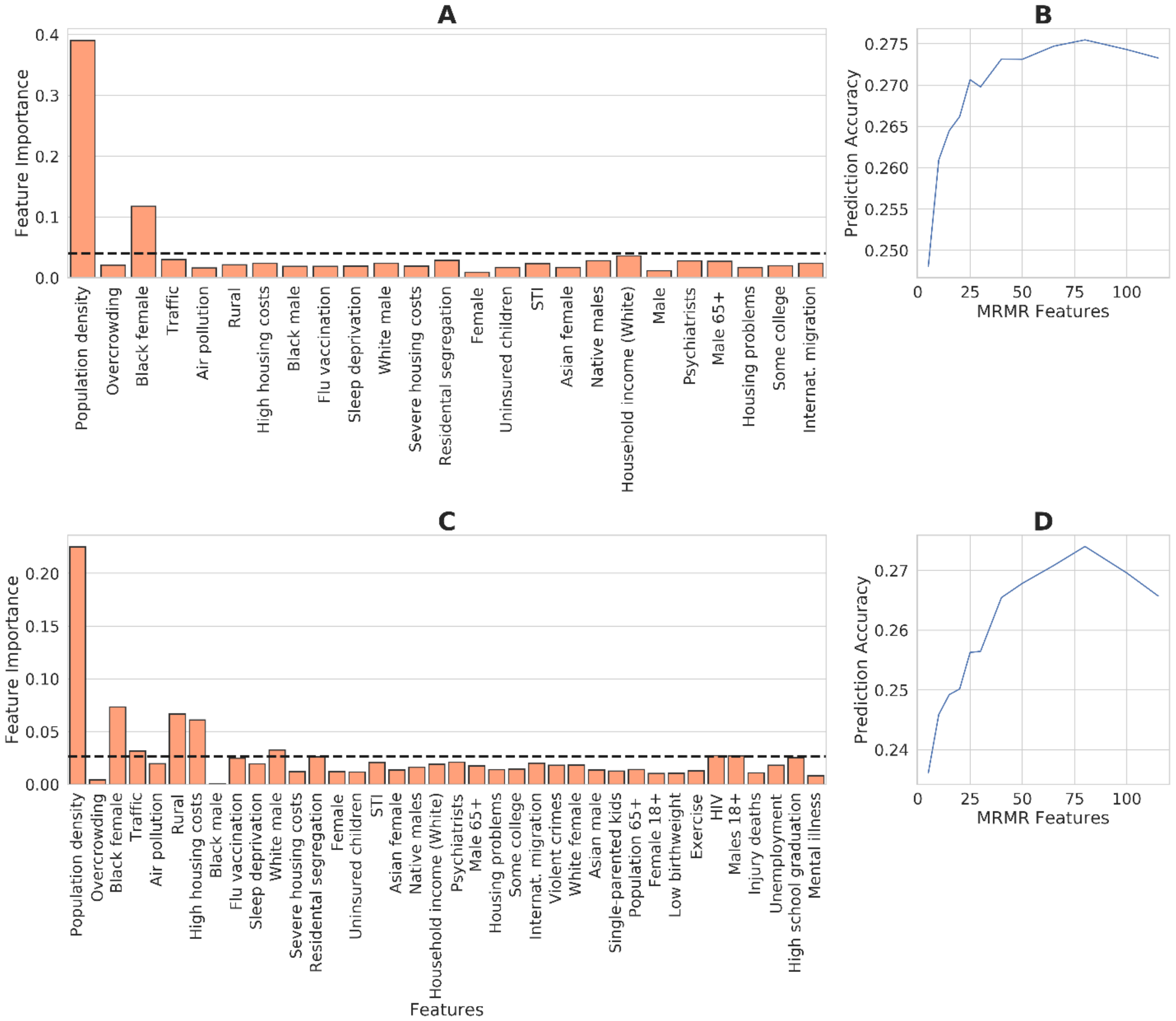

Supplementary Figure 2: Relaxed Random Forest and XGBoost with mRMR feature selection. Feature importance estimates in Relaxed Random Forest (A) and XGBoost (C) regression, Feature selection for Random Forest (B) and XGBoost (D) by mRMR method.
