# Supplementary material for: A large-scale machine learning study of sociodemographic factors contributing to COVID-19 severity
Source: Front Big Data. 2023 Mar 24;6:1038283. doi: 10.3389/fdata.2023.1038283 (PMC10080051; doi:10.3389/fdata.2023.1038283)
Supplement: Supplementary file 15 [file Data_Sheet_15.PDF]

Derivation of m/r

The disease severity measure, m/r, was derived following the methodology introduced in (Markovic et al., 2021). From the SPEIRD modification of the SEIR compartmental model, introduced in (Djordjevic et al., 2021), active cases of the disease (A) can either transfer to the healed (H), with the recovery rate r, or, with the mortality rate m, they become the fatalities (F), which is mathematically represented by the following system of differential equations:

$$\frac{dH}{dt} = rA\,;\ \frac{dF}{dt} = mA \tag{1}$$

that combine into:

$$\frac{dF}{dt} = \frac{m}{r}\frac{dH}{dt} \tag{2}$$

At the end of an epidemiological peak (t = ∞), when the cumulative number of detected cases (D) reaches saturation, and there are no more active cases, the relation between F(∞) and H(∞) is obtained by integrating Eq. (2) from t = 0 to t = ∞:

$$F(\infty) = \frac{m}{r}H(\infty) \tag{3}$$

As the cumulative number of detected cases at any time equals the sum of active cases, healed and fatalities, and the number of active cases at the end of a peak is approximately zero, it follows that:

$$D(\infty) = H(\infty) + F(\infty) \tag{4}$$

By combining Eqs. (3) and (4) we obtain:

$$\frac{m}{r} = \frac{F(\infty)}{D(\infty) - F(\infty)} \tag{5}$$

If we define case fatality rate (CFR) at the end of the peak as the number of fatalities divided by the total number of detected cases ($CFR(\infty) = \frac{F(\infty)}{D(\infty)}$), we get:

$$\frac{m}{r} = \frac{CFR(\infty)}{1 - CFR(\infty)} \tag{6}$$

References:

Djordjevic, M., Rodic, A., Salom, I., Zigic, D., Milicevic, O., Ilic, B., et al. (2021). “Chapter Nine - A systems biology approach to COVID-19 progression in population,” in *Advances in Protein Chemistry and Structural Biology* Proteomics and Systems Biology., eds. R. Donev and T. Karabencheva-Christova (Academic Press), 291–314. doi: 10.1016/bs.apcsb.2021.03.003.

Markovic, S., Rodic, A., Salom, I., Milicevic, O., Djordjevic, M., and Djordjevic, M. (2021). COVID-19 severity determinants inferred through ecological and epidemiological modeling. *One Health* 13, 100355. doi: 10.1016/j.onehlt.2021.100355.
